# Vacuoles, E1 Enzyme, X-linked, Autoinflammatory, and Somatic (VEXAS) Syndrome Presenting With Bicytopenia and Vasculitis

**DOI:** 10.7759/cureus.88083

**Published:** 2025-07-16

**Authors:** José Monge, Kimberly Miranda

**Affiliations:** 1 Hematology, Universidad de Costa Rica, San José, CRI; 2 Hematology, Hospital México, San José, CRI

**Keywords:** auto-inflammatory disease, leukocytoclastic vasculitis (lcv), myelodysplasia, uba1 mutation, vexas syndrome

## Abstract

Vacuoles, E1 enzyme, X-linked, autoinflammatory, and somatic (VEXAS) syndrome is a recently described autoinflammatory condition caused by somatic mutations in the UBA1 gene, typically affecting adult males. Typical clinical features include systemic inflammation, hematologic abnormalities, and various cutaneous manifestations. Due to its recent recognition, the disease poses significant diagnostic and therapeutic challenges. We report the case of a 56-year-old man who presented with recurrent fever, skin lesions consistent with leukocytoclastic vasculitis, macrocytic anemia, and thrombocytopenia. Bone marrow examination revealed dysplastic features and foamy macrophages. Given the suspicion of an autoinflammatory disease, molecular testing using next-generation sequencing was requested, and a somatic mutation in UBA1* *was confirmed*. *This case highlights the importance of considering VEXAS in patients with unexplained systemic inflammation and hematologic abnormalities, and underscores the utility of genetic testing in reaching a definitive diagnosis.

## Introduction

Vacuoles, E1 enzyme, X-linked, autoinflammatory, and somatic (VEXAS) syndrome is a rare disorder that involves a combination of systemic inflammation, hematologic abnormalities, and dermatologic manifestations. This condition is caused by somatic mutations in the UBA1 gene, which encodes an enzyme responsible for protein degradation [1].

The syndrome often presents with a variety of clinical features, including recurrent fevers, skin lesions, cytopenias, and myelodysplastic changes [1]. Although the disease is infrequent, its recognition is becoming increasingly important due to its distinct genetic basis and clinical manifestations, which differentiate it from other inflammatory and hematologic disorders. As more cases are identified, the understanding of the disease pathophysiology, prognosis, and treatment options continues to evolve, with immune-modulating therapies, such as immune modulators, Janus kinase (JAK) inhibitors, and biological therapies, which are showing promise in managing symptoms and improving patient outcomes [2].

This case report describes a middle-aged male patient with no history of chronic diseases, who presented with cutaneous vasculitis, bicytopenia, and an episode of thrombophlebitis. After initial studies by the hematology department, dysplasia was identified. Suspecting an autoinflammatory disease, next-generation sequencing (NGS) was requested, which revealed a mutation in the UBA1 gene.

## Case presentation

A 56-year-old male patient was referred to hematology consultation by his endocrinologist due to normochromic and normocytic anemia with moderate thrombocytopenia observed on routine complete blood count (CBC). Peripheral blood smear described normocytic red blood cells with anisocytosis. He had no symptoms related to anemia or a history of bleeding. The bycitopenia has been documented in previous CBCs for almost a year, but has not been studied by hematology before. 

His personal medical history included the following: diffuse toxic goiter, previously treated with radioactive iodine (I-131) and currently treated with propylthiouracil (PTU) and propranolol; diagnosis of superficial thrombophlebitis one year before, which was treated with Rivaroxaban for six months by a general physician; and generalized purpuric lesions resembling vasculitis on the upper and lower limbs in the past year, for which he was referred by the general physician to dermatology, and skin biopsies were performed. These biopsies reported an acute small-vessel leukocytoclastic vasculitis.

At the moment of his first consultation with the hematologist, the physical examination was unremarkable. There was no splenomegaly or skin lesions, as he received a short course of one month of prednisone prescribed by the dermatologist at a dose of 25 mg daily with a taper.

As part of the complementary studies, deficiencies in iron, vitamin B12, and folic acid were excluded. The erythrocyte sedimentation rate (ESR) was notably elevated (see Table 1), but antinuclear antibodies and serum complement levels showed no abnormalities. Coagulation labs were normal. The antiphospholipid syndrome panel was also negative. Both renal and liver function tests were in the normal range. 

**Table 1:** Diagnostic laboratory values CU - chemiluminescence units *Reference values established by local laboratory.

Parameter	Patient value	Reference range*
Hemoglobin	8.0 g/dL	13-17 g/dL
Platelets	54.0 × 10^3^/µL	150-450 × 10^3^/µL
Leukocytes	3.8 × 10^3^/µL	4-10 × 10^3^/µL
Erythrocyte sedimentation rate (ESR)	124 mm/hour	0-15 mm/hour
Antinuclear antibodies	8 CU	<20 CU
Complement C3	146 mg/dL	75-175 mg/dL
Thyroid-stimulating hormone (TSH)	3.59 mIU/L	0.4-4 mIU/L
C-reactive protein (CRP)	106 mg/L	<10 mg/L

Differential diagnosis included nutritional deficiencies (which were ruled out as mentioned before), chronic diseases such as chronic kidney disease or cirrhosis (both ruled out by labs), and autoimmune disease, ruled out considering the lack of clinical features diagnostic of systemic vasculitis or systemic erythematous lupus (SLE), as well as negative antinuclear antibody (ANA).

Suspecting a primary bone marrow cause of the cytopenias, for example, a bone marrow failure syndrome, peripheral blood flow cytometry was performed to evaluate for paroxysmal nocturnal hemoglobinuria, which was negative. The hematologist proceeded then with bone marrow studies. 

**Figure 1:**
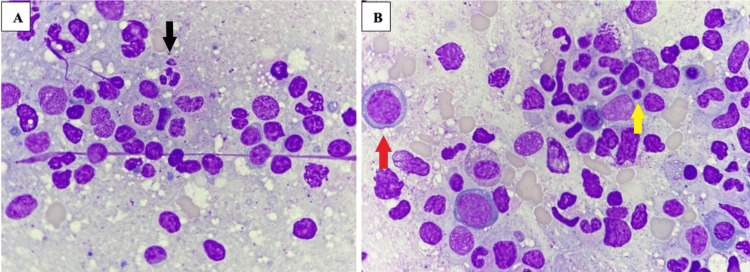
Bone marrow aspirate, high power field A - Hypogranular granulocyte (black arrow). B - Megaloblasts (red arrow) and pseudo-Pelger-Huet cell (yellow arrow).

**Figure 2:**
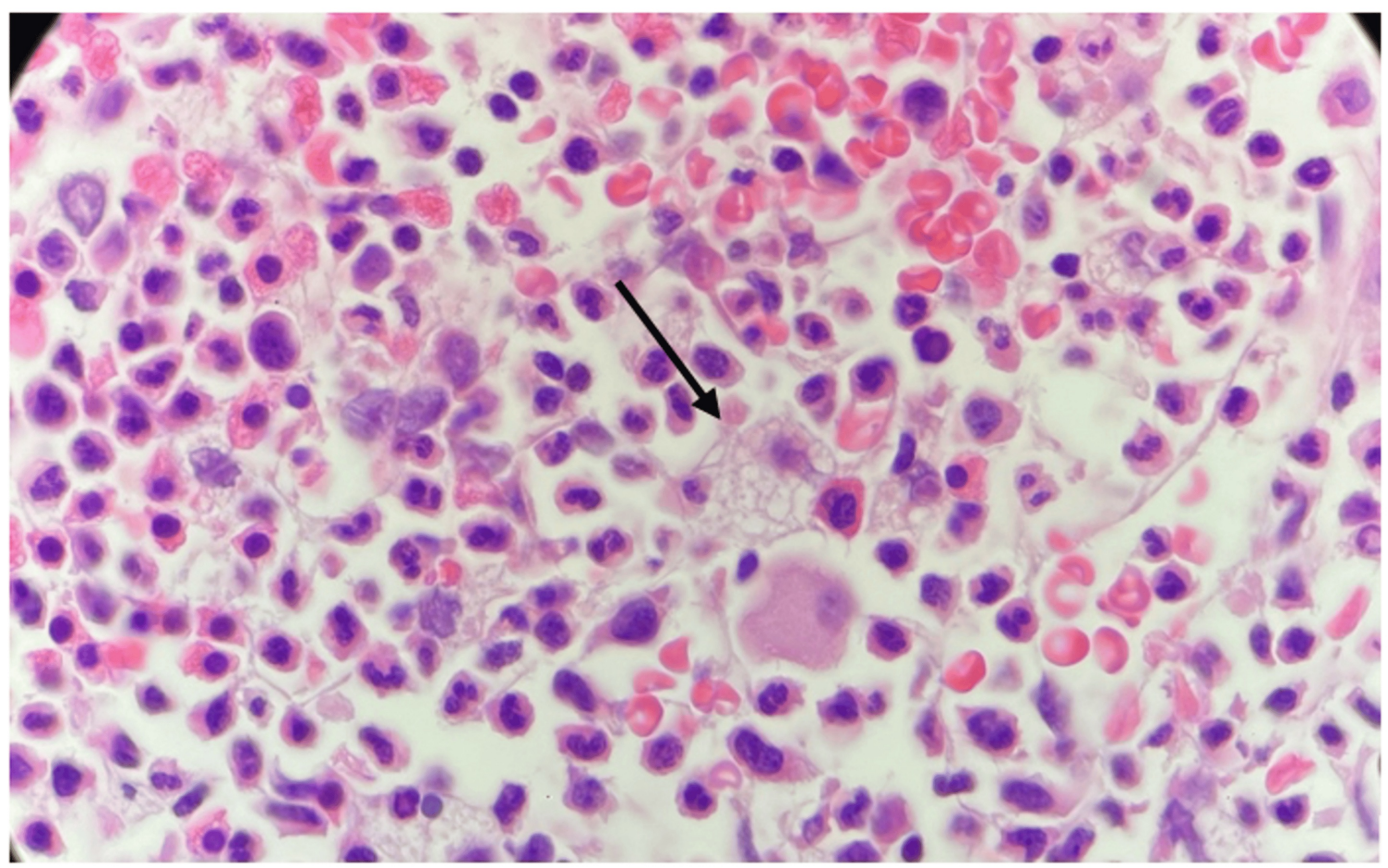
Bone marrow biopsy, high power field (hematoxylin and eosin) A foamy macrophage pointed by a black arrow.

At first, a bone marrow aspirate was performed, which showed an increase in the myeloid series, hypogranular granulocytes (Figure 1A), pseudo-Pelger-Huet anomaly (Figure 1B), and megaloblastic forms in the erythroid series (Figure 1B). Because of inconclusive findings, six months later, a bone marrow biopsy (Figure 2) was obtained as an outpatient. This time, the pathologist’s review described foamy macrophages randomly distributed in the medullary interstitium. During these months, the patient was only taking medications for his chronic conditions.

With the suspicion of a myelodysplastic neoplasm (MDN), a bone marrow flow cytometry was requested, which demonstrated an immunophenotypic aberrance in the pattern of maturation of the neutrophil line, which was in favor of an MDN diagnosis.

Given the past history of leucocytoclastic vasculitis and thrombophlebitis, along with bone marrow findings suggestive of dysplasia, the hematologist suspected an autoinflammatory disease. Therefore, NGS of a bone marrow sample was performed, which identified a somatic mutation in the UBA1 gene, specifically the Met41Val variant.

While awaiting the NGS results as an outpatient, the patient developed a complication due to community-acquired pneumonia caused by Enterobacter cloacae complex with extended-spectrum beta-lactamases, which required invasive mechanical ventilation and norepinephrine due to septic shock. He completed antibiotic treatment with meropenem because of antibiotic resistance. Following the resolution of the infection, corticosteroid therapy was initiated with intravenous methylprednisolone at a dose of 100 mg daily for five days with an overlap to oral prednisone with progressive taper to a dose of 25 mg daily, which was continued for outpatient management. 

During treatment with glucocorticoids, there was no reappearance of skin lesions. However, anemia began to worsen at values below 7 g/dL and required packed red blood cell transfusions, approximately one unit (250 mL) per month. In follow-up outpatient evaluations by Hematology and Immunology, a decision was made to gradually taper the steroid dose and initiate Tocilizumab, which was started without complications at a dose of 162 mg subcutaneously once weekly.

The patient is currently undergoing evaluation for allogeneic bone marrow transplantation as a potential curative treatment for his disease.

## Discussion

VEXAS syndrome is a recently defined condition of great clinical interest, as it represents a primary bone marrow disorder specifically affecting the myeloid and erythroid lineages, leading to severe inflammatory manifestations, cytopenias, and thrombotic complications [2].

Approximately half of the reported cases are characterized by the Met41Thr substitution in the UBA1 gene, while the patient described in this case belongs to the 21% of variants carrying the Met41Val substitution [3].

The pathophysiology is driven by the occurrence of UBA1 mutations, which appear to exert positive selection pressure on myeloid and erythroid progenitors, in contrast to the lymphoid lineage, in which negative clonal selection occurs. This phenomenon correlates with the lymphopenia commonly observed in these patients [4].

Fever, weight loss, and fatigue are frequent presenting symptoms. Macrocytic anemia has been reported as a universal finding, while thrombocytopenia is observed in up to 50% of cases [5]. Morphologic changes such as hypogranularity and pseudo-Pelger-Huet anomalies can also be identified [6].

Regarding myelodysplastic syndrome (MDS), some diagnostic criteria were met in this case based on the presence of anemia (hemoglobin <10 g/dL) and thrombocytopenia (platelets <100 × 10⁹/L) [7]. Nonetheless, dysplasia was identified in more than 10% of granulocytic cells. However, one of the characteristic histologic patterns of VEXAS includes myeloid hyperplasia, increased megakaryocytes, and dysplastic changes in some lineages, but often affecting less than 10% of the evaluated cells [8]. 

It has been proposed that UBA1 mutations may act as a driver of MDS and could eventually be incorporated into molecular panels as a diagnostic criterion for this disease [2].

This case also illustrates the most frequently reported type of vasculitis in VEXAS syndrome, which is leukocytoclastic vasculitis. The main clinical finding is purpura, and it has been described that vasculitis affecting larger vessels tends to be more refractory to steroids and biological agents [9]. In this case, the patient presented with small-vessel vasculitis and had a favorable response to initial therapy with steroids and immune modulation.

Another key clinical finding in this patient was the history of thrombophlebitis. Thrombotic complications may occur early in the disease course, with a prevalence of 44% in previous cohorts [6]. Most events involve venous thrombosis. Although the precise mechanism is unclear, one explanation is that there is increased factor VIII activity, in association with elevated C-reactive protein levels, which promotes fibrin formation and consumption of natural anticoagulants [3].

Patients commonly respond initially to glucocorticoids, typically at doses of 20-40 mg of prednisone daily (as in this case). However, disease flares are frequent upon dose tapering [5].

Supportive care with antimicrobial and antiviral prophylaxis is essential, as is thromboprophylaxis during high-risk periods. Anticoagulation is mandatory in the setting of thrombosis [2]. In this patient, anticoagulation with Rivaroxaban at a dose of 20 mg daily was administered for six months without recurrence of thrombosis.

Given the elevated circulating levels of interleukin-6 (IL-6) observed in VEXAS, Tocilizumab has shown modest efficacy in disease control, particularly when combined with low-dose steroids [10]. Other therapies used include methotrexate, JAK inhibitors, and calcineurin inhibitors [2].

In a previous report of 83 patients, an overall mortality rate of 25% was documented. The Met41Val mutation and transfusion dependence were identified as risk factors for poor prognosis [11]. Based on these findings, some authors recommend considering allogeneic bone marrow transplantation in these high-risk patients, although current experience remains limited to small case series [3].

In this particular case, Tocilizumab was prescribed with the aim of reducing long-term steroid dependence, and the patient was also enrolled in an allogeneic bone marrow transplantation program, given the presence of a high-risk genetic variant.

## Conclusions

VEXAS syndrome is an emerging autoinflammatory condition with a highly variable clinical presentation that often overlaps with other rheumatologic, dermatologic, and hematologic diseases, making diagnosis challenging. In this case, the combination of systemic inflammation, cutaneous vasculitis, macrocytic anemia, and thrombocytopenia raised suspicion for an underlying clonal disorder. The identification of a somatic UBA1 mutation through genetic testing was essential to confirm the diagnosis. It is of utmost importance to consider the possibility of VEXAS syndrome in patients with unexplained inflammatory symptoms and hematologic abnormalities, as early recognition is crucial to make management decisions and establish a prognosis. Given the lack of standardized treatment protocols, a multidisciplinary approach remains key in tailoring care to the individual patient’s manifestations. As the medical community continues to report these cases, there will be a better understanding of the disease course, and it may help refine therapeutic strategies in the future.
